# The Role of IL-6 and TNF-α as Early Biomarkers in the Prediction and Diagnosis of Gestational Diabetes Mellitus

**DOI:** 10.3390/biomedicines13071627

**Published:** 2025-07-02

**Authors:** Antonia Varthaliti, Vasilios Lygizos, Maria Fanaki, Vasilios Pergialiotis, Angeliki Papapanagiotou, Kalliopi Pappa, Marianna Theodora, Maria Anastasia Daskalaki, Panos Antsaklis, George Daskalakis

**Affiliations:** 1 1st Department of Obstetrics and Gynecology, “Alexandra” General Hospital, National and Kapodistrian University of Athens, 80 Vasilissis Sofias Avenue, 11528 Athens, Greece; vlygizos@gmail.com (V.L.); maria.fanaki@gmail.com (M.F.); pergialiotis@hotmail.com (V.P.); kalliopi.pappa20@gmail.com (K.P.); martheodr@gmail.com (M.T.); anastasia.daskalaki00@gmail.com (M.A.D.); panosant@gmail.com (P.A.); gdaskalakis@yahoo.com (G.D.); 2 Department of Biological Chemistry, Medical School, National and Kapodistrian University of Athens, 15772 Athens, Greece; agpana@med.uoa.gr

**Keywords:** gestational diabetes mellitus, diagnosis, biomarkers, IL-6, TNF-α, pregnancy

## Abstract

Gestational diabetes mellitus (GDM) occurs in approximately 9–25% of pregnancies and, if left undiagnosed or inadequately controlled, can lead to adverse outcomes for both the mother and the fetus, short and long term. GDM is characterized by glucose intolerance with onset or first recognition during pregnancy and is a multifactorial condition with a pathophysiology that remains incompletely understood. It is strongly associated with a chronic low-grade inflammatory state that contributes to insulin resistance, a hallmark of GDM pathogenesis. Among the fundamental pro-inflammatory cytokines implicated in this process, TNF-α and IL-6 play central roles. TNF-α is a cytokine primarily secreted by activated macrophages, as well as by adipocytes in the context of obesity. Many studies have shown that its levels are elevated in pregnant women with GDM compared to normoglycemic pregnant individuals. IL-6 is another pro-inflammatory cytokine secreted by immune cells, adipose tissue, and the placenta. It is found in higher concentrations in the maternal circulation during pregnancies complicated by GDM. Both TNF-α and IL-6 act synergistically to perpetuate a pro-inflammatory intrauterine environment. Their combined effects exacerbate insulin resistance and may impair pancreatic β-cell compensation during pregnancy, facilitating the onset of GDM in genetically or metabolically susceptible individuals. Recent research has identified various maternal serum biomarkers, such as TNF-α and IL-6, that may hold promise for the early detection of GDM. The aim of our study is to evaluate whether TNF-α and IL-6 can be used as diagnostic tools for the early diagnosis of GDM, allowing for timely intervention and reducing the risk of associated maternal and fetal complications.

## 1. Introduction

Gestational diabetes mellitus (GDM) is one of the most common metabolic disorders of pregnancy, affecting approximately 7–14% pregnancies worldwide [1]. It is characterized by glucose intolerance with onset or first diagnosis during pregnancy and is associated with significant short- and long-term complications for both the mother and the fetus. Maternal risks include preeclampsia, cesarean delivery, and progression to type 2 diabetes mellitus (T2DM), while it may cause fetal complications such as macrosomia, preterm delivery or even stillbirth, neonatal hypoglycemia, and increased lifetime risk of obesity and T2DM.

The current gold-standard for the diagnosis of GDM is the oral glucose tolerance test (OGTT) that is typically performed between 24–28 weeks of gestation. However, OGTT has several limitations, which include patient inconvenience, fasting requirements, poor reproducibility, and its relatively late administration in pregnancy. Thus, early intervention is not possible, and it may cause irreversible complications. As a result, there is a growing interest in identifying early, reliable biomarkers that reflect the pathophysiological processes leading to GDM and can lead to early diagnosis [2].

Chronic inflammation is recognized to play a critical role in the pathogenesis of insulin resistance and GDM. Among various inflammatory mediators, interleukin-6 (IL-6) and tumor necrosis factor-alpha (TNF-α) have been extensively studied for their contributions to both systemic insulin resistance and the metabolic alterations that occur during pregnancy. Both cytokines are secreted by adipose tissue, placental trophoblasts, and immune cells, and have been found to be elevated in women who later develop GDM. Their role in disrupting insulin signaling pathways and changing glucose homeostasis suggest their potential as early biomarkers for GDM prediction and diagnosis [3].

This narrative review aims to present the current evidence in the role of IL-6 and TNF-α as biomarkers for early diagnosis of GDM. We will examine their physiological relevance, evidence from observational and prospective studies, potential integration into clinical screening algorithms, and future directions for research.

## 2. Pathophysiology of Gestational Diabetes Mellitus

Gestational diabetes mellitus (GDM) is a complex metabolic condition that arises from the interaction between insulin resistance and insufficient pancreatic β-cell compensation during pregnancy. Although GDM was considered a condition that develops in the late second trimester of pregnancy, current research indicates that metabolic dysfunction may begin even before conception, emphasizing the importance of early physiological changes in its pathogenesis.

### 2.1. Insulin Resistance and β-Cell Dysfunction

**Figure 1:**
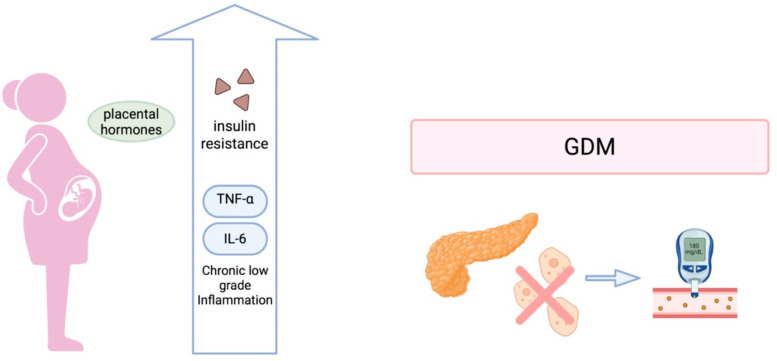
Insulin resistance and GDM during pregnancy. Created in BioRender. Varthaliti, A. (2025) https://BioRender.com/puwn1w6.

Pregnancy is characterized by progressive insulin resistance, as shown in Figure 1, primarily affected by placental hormones such as human placental lactogen, progesterone, cortisol, and placental growth hormone, increasing by approximately 40–50% as gestation advances. These adaptations are essential to facilitate nutrient transfer to the fetus. In healthy pregnancies, insulin secretion rises to adapt to the increased insulin resistance. However, in GDM, this mechanism is inadequate due to β-cell dysfunction, resulting in maternal hyperglycemia. The degree and timing of these malfunctions vary, leading to distinct metabolic phenotypes of GDM, including insulin-resistant, insulin-deficient, and mixed subtypes [4].

### 2.2. Inflammation and Cytokine-Mediated Insulin Resistance

**Figure 2:**
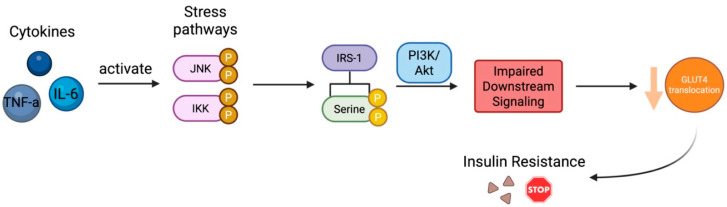
Cytokine-Mediated Insulin Resistance. Created in BioRender. Varthaliti, A. (2025) https://BioRender.com/bsccp43.

Emerging evidence identifies chronic low-grade inflammation as a central mechanism in the pathogenesis of GDM. Pro-inflammatory cytokines, particularly IL-6 and TNF-α, play critical roles in impairing insulin signaling. These cytokines, produced by adipose tissue, placenta, and immune cells, activate intracellular stress pathways such as c-Jun N-terminal kinase (JNK) and inhibitor of kappa B kinase (IKK), leading to serine phosphorylation of insulin receptor substrate-1 (IRS-1) and impaired downstream signaling via the PI3K/Akt pathway. Consequently, glucose transporter 4 (GLUT4) translocation is reduced, resulting in diminished glucose uptake and systemic insulin resistance (Figure 2).

TNF-α disrupts insulin signaling by promoting serine phosphorylation of IRS proteins and impairing adiponectin signaling. IL-6, in turn, contributes to hepatic glucose production and suppresses insulin sensitivity in peripheral tissues. These inflammatory mediators are elevated in early pregnancy in women who subsequently develop GDM, underscoring their potential utility as early biomarkers for prediction and diagnosis [5].

### 2.3. Placental and Fetal Contributions

The placenta exerts a profound influence on maternal metabolic adaptation through secretion of hormones, cytokines, and exosomes. In GDM, alterations in placental function may precede clinical hyperglycemia. Trophoblastic mitochondrial dysfunction and oxidative stress can impair insulin sensitivity and placental nutrient transport. Fetal hyperinsulinemia, induced by maternal hyperglycemia, promotes excessive nutrient uptake and contributes to fetal overgrowth, while also modifying placental signaling loops in a feed-forward manner [4].

### 2.4. Non-Cytokine Mediators Involved in Glucose Regulation

Recent studies have demonstrated that except for the classical inflammatory cytokines, like TNF-α and IL-6, neurotrophic and gut-derived factors also contribute to the glucose regulation during pregnancy and may play a role in the development of GDM. Brain-derived neurotrophic factor (BDNF) seems to decrease in women with GDM [6,7]. This might reflect a change of insulin sensitivity and metabolic central regulation. It is well known that GLP-1 receptor signaling is essential for insulin secretion, stimulated by glucose, and its function may be exacerbated in GDM due to cytokines like TNF-α, further contributing to β-cell dysfunction [8]. Furthermore, insulin secretion induces serotonin production by pancreatic β-cells and acetylcholine production by parasympathetic neurons, which are affected by hormonal changes and inflammatory signals during pregnancy [9,10,11]. Serotonin is produced by β-cells and enhances insulin secretion, particularly during pregnancy. Dysregulated serotonin synthesis or signaling may impair this adaptive mechanism, contributing to β-cell dysfunction in GDM. Similarly, acetylcholine, acting through muscarinic receptors on β-cells, facilitates glucose-stimulated insulin release. Inflammation-induced impairment of vagal tone or cholinergic signaling could exacerbate insulin secretory defects in GDM.

Another important factor, the duodenal mucosa is a site for incretin hormone secretion, such as GLP-1, but also harbors enteroendocrine cells and immune components that respond to metabolic and inflammatory process. This mucosal dysregulation, including altered microbiota and cytokine expression, may impair gut–pancreas crosstalk, and as a result, GDM. All these findings support the implication of multiple factors in the pathophysiology of GDM that includes immune–neuroendocrine interactions at both systemic and gastrointestinal levels [12].

Gestational diabetes mellitus (GDM) is increasingly recognized as a systemic condition involving not only insulin resistance and glucose dysregulation but also a complex interplay between inflammatory, neuroendocrine, and metabolic factors. While pro-inflammatory cytokines such as TNF-α and IL-6 have been extensively studied in the context of GDM, a broader perspective reveals significant contributions from anti-inflammatory cytokines, adipokines, and neuropeptides.

Anti-inflammatory cytokines such as adiponectin, ghrelin, irisin, and vaspin serve to counterbalance the damaging effects of chronic low-grade inflammation in GDM. According to the study by Lis-Kuberka et al. [13], the levels of irisin and vaspin in colostrum were significantly modulated depending on the severity of maternal hyperglycemia. Specifically, higher vaspin levels were observed in insulin-treated GDM mothers compared to normoglycemic women, suggesting a possible compensatory insulin-sensitizing role in more severe metabolic dysregulation. Irisin is a myokine with metabolic regulatory functions and showed significant differences between diet-treated and insulin-treated mothers, possibly reflecting a difference in metabolic adaptation or insulin sensitivity [13]. These findings underscore the role of anti-inflammatory adipokines as potential modulators of glucose metabolism and immune response in GDM.

Ansari-Lari et al. highlighted that leptin levels were elevated in women with GDM, whereas TNF-α levels were paradoxically lower [14]. These findings suggest complex regulatory feedback between inflammatory cytokines and metabolic hormones in GDM. Leptin, primarily secreted by adipocytes, is involved in appetite regulation, insulin sensitivity, and immune modulation, and its dysregulation may reflect both inflammatory status and altered energy homeostasis in GDM.

Current evidence supports a multifactorial pathogenesis of GDM involving pro- and anti-inflammatory imbalance, adipokine dysregulation, impaired gut–brain–pancreas axis, and neuroimmune interactions. These insights justify a revised conceptual model of GDM not as a purely metabolic or inflammatory disorder, but as a neuroimmune metabolic condition with implications for maternal and neonatal outcomes. Future research should aim to integrate these diverse pathways into predictive and therapeutic strategies.

## 3. Biological Functions of Interleukin-6 and TNF-α

Chronic low-grade inflammation is associated with the pathogenesis of GDM, connecting adipose-derived and placental cytokines to metabolic dysregulation [15,16]. In particular, two of the pro-inflammatory cytokines, IL-6 and TNF-α, are the main cytokines that have been extensively studied as potential early biomarkers for predicting and diagnosing GDM. Both IL-6 and TNF-α are elevated in obesity and metabolic syndrome and can induce insulin resistance by disrupting insulin signaling pathways [15,17].

TNF-α and IL-6 are key pro-inflammatory cytokines that are consistently elevated in insulin resistance and GDM. Their involvement in impairing insulin signaling and counteracting insulin’s anti-inflammatory effects positions them as potential mediators of insulin resistance. Under normal conditions, insulin reduces the generation of reactive oxygen species (ROS) by mononuclear cells; however, this protective effect is compromised in the presence of TNF-α and IL-6 [18,19,20]. These cytokines originate from both maternal and placental immune systems, which typically coordinate to maintain fetal tolerance. In GDM, however, the maternal immune environment remains skewed toward a pro-inflammatory state, with persistently elevated levels of TNF-α and IL-6 [2,21].

TNF-α is a pro-inflammatory cytokine primarily produced by activated monocytes and macrophages, and it plays an important role in inflammatory response [15]. It contributes to insulin resistance by inducing serine phosphorylation of IRS-1, which disrupts its interaction with the insulin receptor [22,23,24]. This leads to impairment of the insulin receptor tyrosine kinase activity and downstream signaling necessary for glucose uptake. Specifically, TNF-α blunts the normal action of insulin at the cellular level, leading to reduced glucose transport into cells. Studies have shown that TNF-α can activate pathways (like sphingolipid and NF-κB signaling) that impair insulin receptor autophosphorylation and promote insulin resistance in both adipocytes and skeletal muscle [17]. In GDM, TNF-α is considered an independent risk factor for dysglycemia due to this ability to disrupt insulin action, leading to increased insulin resistance. Additionally, TNF-α has been shown to suppress the expression of the adiponectin gene in human adipocytes [24,25]. Since adiponectin is an insulin-sensitizing hormone, reduced levels because of heightened TNF-α activity may further increase insulin resistance in GDM. GDM placentas have demonstrated increased TNF-α expression and release [26], which can spill into maternal circulation.

IL-6 is a pro-inflammatory cytokine and is secreted by immune cells, endothelial cells, and primarily by monocytes and macrophages within adipose tissue and exerts broad effects on glucose metabolism. IL-6 is involved in a wide range of biological functions, including regulation of acute phase protein responses, modulation of B and T lymphocyte activity, alteration of blood-brain barrier permeability, promotion of synovial inflammation, influence on blood cell formation, and contributions to fetal development. It influences pancreatic islet β-cell function, where it has been shown to enhance insulin secretion under certain conditions [27]. Furthermore, IL-6 promotes hepatic glucose production by stimulating gluconeogenesis and glycogenolysis, and it impairs insulin action in peripheral tissues. Elevated IL-6 levels are commonly observed in obesity and have been linked to altered insulin sensitivity in hepatic tissue and pancreatic β-cells, thereby contributing to the development of insulin resistance [28]. During pregnancy, placental production of IL-6 is believed to induce a chronic inflammatory state within adipose tissue, further exacerbating the physiological insulin resistance characteristic of gestation [29].

In GDM, there is placental expression of pro-inflammatory cytokines, especially TNF-α and IL-6 [18], which is associated with elevated leptin mRNA expression in the placenta. Leptin, in turn, has been shown to enhance the secretion of TNF-α and IL-6 by monocytes [27], thereby reinforcing the inflammatory response. Additionally, the hyperinsulinemic state characteristic of GDM may further promote leptin production, which exacerbates inflammation through this feedback mechanism. This bidirectional interaction establishes a self-perpetuating cycle that sustains the inflammatory state and aggravates insulin resistance.

Recent studies have shown that proinflammatory cytokines may downregulate the expression or affect signaling of the GLP-1 receptor (GLP-1R) in pancreatic β-cells, thereby limiting the insulinotropic action of GLP-1. This mechanism is particularly important in the case of gestational diabetes mellitus, where mild chronic inflammation is observed. Increased levels of TNF-α, which are frequently found in pregnant women with GDM, have been shown to suppress GLP-1R expression. As a result, this leads to reduced insulin secretion in response to glucose. This phenomenon affects not only insulin sensitivity, but also the ability of β-cells to adequately respond to the increased metabolic demands of pregnancy. This interaction highlights the dual role of inflammation in the pathogenesis of GDM, both through the induction of insulin resistance and through the dysfunction of compensatory mechanisms of insulin secretion [30].

TNF-α and IL-6 are expressed in placental tissues and affect trophoblast proliferation, apoptosis, and invasion [31]. Increased levels of TNF-α and IL-6 may contribute to placental dysfunction, impaired nutrient transport, and inflammatory disruption of the utero-fetal interface, factors that have been implicated in the pathogenesis of gestational diabetes mellitus [32].

## 4. Predictive Value, Integration into Screening, and Future Perspectives

There are multiple clinical studies that have examined maternal IL-6 and TNF-α concentrations during pregnancy in relation to GDM development, as shown in Table 1. There is evidence that women who develop GDM have higher circulating IL-6 levels, even before clinical diagnosis. A study by Hosseini et al. found that higher maternal IL-6 levels are significantly associated with increased risk of GDM (pooled OR ~1.35, 95%CI 1.05–1.73) [33]. Notably, this association was evident even in early gestation. Particularly, IL-6 measured at 12–15 weeks was significantly elevated in women that later developed GDM compared to those who did not [33]. Similarly, Gennaro et al. reported that first-trimester IL-6 levels could help identify women at high risk for GDM [26]. Furthermore, Abell et al. observed that women who eventually developed GDM had increased IL-6 in the first trimester, suggesting an early pro-inflammatory state preceding hyperglycemia [26]. These findings support IL-6 as a candidate early biomarker of GDM, detectable well before the typical 24–28 week GDM screening period. By the time GDM is diagnosed, usually late in the second trimester, IL-6 increase becomes even more prominent. Case–control studies across diverse populations have shown significantly higher IL-6 in GDM patients compared to normoglycemic pregnant controls at mid-pregnancy. Specifically, the study by Siddiqui et al. reported that serum IL-6 levels in women with GDM were significantly elevated versus controls (mean IL-6 GDM 8.21 pg/mL vs. controls 5.23 pg/mL, *p* < 0.05) [34]. IL-6 emerged as an inflammatory mediator “augmented” in GDM, implicating inflammation in GDM pathophysiology [34]. Wang et al. noted a similar trend that GDM pregnant women had markedly higher IL-6 levels than those with normal glucose tolerance, alongside elevated TNF-α and C-reactive protein, reflecting a heightened inflammatory state [35]. Obesity is not clearly and fully associated with IL-6 and, in fact, GDM-related IL-6 elevation appears independent of BMI in some analyses. In other words, even after accounting for pre-pregnancy adiposity, GDM itself is linked to higher IL-6, likely due to pregnancy-specific inflammatory changes, including placental cytokine production. The placenta can produce IL-6 in significant amounts, and women with GDM show increased placental IL-6 expression compared to euglycemic pregnancies [26]. This placental contribution may sustain high circulating IL-6 late in gestation, further aggravating insulin resistance and hyperglycemia in GDM.

The literature about the correlation between TNF-α levels in early gestation and subsequent GDM is somewhat divergent. Many studies indicate that TNF-α is higher (or trends higher) in women who will develop GDM, even in the first or early second trimester, reflecting a preexisting pro-inflammatory state. Hosseini et al. found that elevated circulating TNF-α was significantly associated with increased GDM risk (pooled OR ~1.28, 95%CI 1.01–1.62). They reported that early/mid-pregnancy TNF-α levels were already higher in women who went on to have GDM compared to controls [33]. For example, in some cohorts, women in the highest tertiles of TNF-α at 15 weeks had greater odds of GDM than those in lower tertiles. These findings support TNF-α as a potential early biomarker. In fact, Tenenbaum-Gavish et al. included TNF-α in a first-trimester predictive model. Although on its own, first-trimester TNF-α was on average lower in GDM cases, the combination of TNF-α with other markers substantially improved prediction of GDM, achieving an AUC of 0.95 (89% detection at 10% false-positive rate) in their dataset [38]. This suggests that, under certain adjustments and in multivariate context, TNF-α carries predictive signal for GDM.

By the time of GDM diagnosis (24–28 weeks), TNF-α is generally found to be elevated in GDM patients. Wei et al. reported that circulating TNF-α was highly expressed in GDM patients compared to healthy pregnant controls [15]. They identified a TNF-α threshold of 5.32 ng/L that distinguished GDM, with high sensitivity and specificity by ROC analysis [15]. Additionally, Zhang et al. conducted a prospective study of 280 women and concluded that the distribution frequency of the *TNF-α-857CT* genotype was notably higher in women with GDM pregnancies compared to those with healthy pregnancies, with an odds ratio of 3.316 (95%CI: 1.092–8.304, *p* = 0.025). Women with GDM were also characterized by older maternal age, higher body mass index (BMI), increased nulliparity, and a greater history of type 2 diabetes mellitus and previous GDM relative to women with healthy pregnancies (*p* < 0.05). Furthermore, inflammatory biomarkers in serum, including hs-CRP, IL-6, IL-8, and the IL-6/IL-10 ratio, as well as placental markers such as NF-κB, IL-6, IL-8, the IL-6/IL-10 ratio, IL-1β, and TNF-α, were found to be significantly elevated (*p* < 0.05) in women with GDM compared to those with healthy pregnancies. Likewise, Mohammed et al. observed significantly higher TNF-α levels in women with GDM at 24–28 weeks compared to normoglycemic pregnant women [40]. This focused on insulin resistance and it found that GDM women had greater insulin resistance (IR) (mean Homeostatic Model Assessment of Insulin Resistance (HOMA-IR) ~3.14) than controls (~2.89), and importantly, TNF-α levels positively correlated with HOMA-IR (r = 0.49, *p* < 0.05) [40]. In multivariate analysis, TNF-α emerged as an independent predictor of insulin resistance in GDM, even after adjusting for age and BMI [40]. Such findings reinforce that TNF-α is not only elevated in GDM but is directly linked to the severity of insulin resistance, a core defect in GDM. Furthermore, Catalano et al. previously showed that higher TNF-α in pregnant women is associated with lower insulin sensitivity and proposed TNF-α as a key factor in gestational IR [17]. Nevertheless, certain findings remain ambiguous or inconsistent across various study populations. A prospective study by Gueuvoghlanian-Silva et al. involving 248 women observed no significant correlation between in vitro levels of IL-6 and TNF-α in the GDM group compared to healthy controls [42]. In another recent systematic review by Gomes et al., it was indicated that while TNF-α concentrations were marginally higher in individuals with GDM compared to control subjects, the difference was not statistically significant [43]. A recent pilot study by Muthuraman et al. unexpectedly found no significant difference in serum TNF-α between GDM and non-GDM mothers at 24–28 weeks (median TNF ~12.4 pg/mL in GDM vs. 14.6 pg/mL in controls, *p* > 0.05) [37]. The GDM group even had a slightly lower median TNF-α. Though underpowered (n ≈ 24 per group), this study cautions that TNF-α levels can vary and might not always rise in GDM, possibly due to ethnic or methodological differences. Thus, while TNF-α is a promising biomarker, it may need to be interpreted in context. Current evidence supports that elevated TNF-α early in pregnancy signals higher GDM risk, aligning with the pathophysiological role of TNF-α in insulin resistance. Nevertheless, incorporating TNF-α into clinical prediction models will require consensus on timing (e.g., first visit measurements), standardized assays, and cutoff definitions to balance sensitivity and specificity.

IL-6 and TNF-α can be included in biomarker panels for GDM for various reasons. These cytokines may serve as early indicators of GDM, particularly in high-risk pregnancies, such as those in women with obesity or a family history of diabetes. Elevated concentrations of these markers could help in identifying pregnant women at an increased risk of developing GDM even earlier than the traditional glucose testing. Additionally, the measurement of cytokine levels throughout pregnancy may facilitate the monitoring of insulin resistance and inflammatory processes, which could inform more personalized treatment and management approaches. However, there are several challenges to consider, including the variability in cytokine levels. These levels can fluctuate due to factors such as the stage of pregnancy, individual immune responses, and the presence of comorbid conditions [44].

However, increased levels of IL-6 and TNF-α are not specific to GDM and they may also increase in inflammatory conditions. As a result, the interpretation of these cytokines should be carefully made and ideally with clinical assessment. Also, further research should be conducted in order to evaluate the levels of these cytokines longitudinally during pregnancy and whether pregnancy alone affects these levels. Also, further maternal characteristics that may affect IL-6 and TNF-α should be taken into consideration in the interpretation of the results.

Incorporating IL-6 and TNF-α into personalized risk stratification for gestational diabetes offers significant insights into the inflammatory and metabolic mechanisms underlying the condition. Tailored monitoring and lifestyle interventions, such as weight management and physical activity, can be provided to women identified as being at elevated risk based on their TNF-α or IL-6 levels. This approach has the potential to mitigate disease severity or delay its onset. Women with elevated levels of TNF-α or IL-6 can be classified as high-risk and prioritized for more frequent screenings (e.g., oral glucose tolerance test (OGTT), insulin sensitivity assessments), while those with lower levels may require less intensive monitoring. This strategy enhances the efficiency of healthcare resource allocation and optimizes patient care.

Maternal demographic and metabolic factors, such as age, body mass index (BMI), ethnicity, family history of diabetes, and pre-pregnancy weight, have long been established as traditional risk factors for gestational diabetes mellitus (GDM). The integration of inflammatory cytokines, particularly IL-6 and TNF-α, into this existing framework enables a more comprehensive and individualized approach to risk assessment and management. Advanced maternal age, particularly in women over 35 years, is associated with an elevated risk of GDM [45]. This demographic group often exhibits higher levels of IL-6 and TNF-α, reflecting age-related chronic inflammation and metabolic dysfunction [46]. Similarly, certain ethnic populations, including Hispanic, African American, and Asian groups, are at an increased risk of GDM [47], with evidence suggesting ethnic variations in cytokine profiles, including IL-6 and TNF-α, which may partly explain these disparities [48]. A family history of type 2 diabetes or GDM is another significant risk factor, as it indicates a genetic predisposition to insulin resistance, potentially correlating with elevated IL-6 and TNF-α levels [49]. Obesity, one of the most prominent risk factors for GDM, is also linked to increased levels of these inflammatory markers due to the inflammatory state induced by excessive adiposity. When IL-6 and TNF-α levels are measured alongside BMI, clinicians can gain a clearer understanding of the degree of insulin resistance and adjust interventions accordingly to improve metabolic outcomes [50]. Furthermore, women with pre-existing insulin resistance or conditions such as polycystic ovary syndrome (PCOS) are more vulnerable to GDM, and the inclusion of IL-6 and TNF-α, in conjunction with other metabolic markers such as HOMA-IR [51], provides a more accurate and nuanced assessment of a woman’s metabolic health [52]. Fasting blood glucose levels, HbA1c, and results from an OGTT are standard tools in GDM diagnosis. Standard diagnostic tests, including fasting blood glucose levels, HbA1c, and the OGTT, remain essential for GDM diagnosis. However, when abnormal glucose test results are accompanied by elevated IL-6 and TNF-α levels, they may indicate more severe insulin resistance, warranting earlier intervention or more intensive monitoring. This integrated approach ultimately offers a more personalized and precise strategy for GDM risk assessment and management.

By integrating measurements of IL-6 and TNF-α with maternal demographic and metabolic data, healthcare providers can enhance the accuracy of predicting the risk of GDM and refine both preventive and therapeutic strategies. For women identified as high-risk, personalized interventions, such as modifications to diet, exercise routines, or pharmacological treatments (e.g., metformin or insulin), can be introduced at earlier stages of pregnancy, before the onset of the irreversible damage for the mother and the fetus. As gestation progresses, continuous monitoring of IL-6 and TNF-α levels, in conjunction with glucose measurements, allows clinicians to assess the progression of insulin resistance and inflammation, facilitating timely adjustments in care. This approach, which emphasizes personalized treatment, enables at-risk women to receive interventions that reduce the severity of GDM, minimize the likelihood of complications such as macrosomia, preeclampsia, and cesarean delivery, and promote long-term maternal health, including a reduced future risk of type 2 diabetes.

## 5. Limitations

Despite the growing evidence supporting the involvement of IL-6 and TNF-α in the pathogenesis of GDM, several limitations must be acknowledged when considering their potential as biomarkers for early diagnosis. First, the existing literature is notable for its considerable heterogeneity in study designs, including differences in patient characteristics and demographics, diagnostic criteria for GDM, and gestational age at the biomarker assessment. This variability limits the ability to draw consistent conclusions or perform meaningful meta-analyses.

Furthermore, there is still no consensus on gestational age-specific reference ranges or clinically validated cut-off values for IL-6 and TNF-α that can accurately predict GDM. Most studies report relative differences in cytokine concentrations between cases and controls, without defining thresholds that can be implemented in clinical screening algorithms. Additionally, there is variability in assay methods used for cytokine quantification. The sensitivity and specificity of methods such as multiplex platforms and enzyme-linked immunosorbent assay (ELISA) vary, which could lead to discrepancies in the reporting of cytokine levels between studies.

Another important limitation is the influence of confounding factors such as maternal body mass index, chronic low-grade inflammation, dietary habits, ethnicity, and stress, all of which can independently modulate circulating cytokine levels. As such, elevated IL-6 or TNF-α concentrations in early pregnancy may not exclusively reflect emerging glycemic dysregulation but rather a combination of overlapping metabolic and immunologic processes. Elevated IL-6 and TNF-α levels are not exclusive to GDM and may also be observed in other conditions, such as infections and autoimmune diseases [53], and also pregnancy-related conditions such as preeclampsia [54] and preterm birth [55]. As a result, these cytokines may not provide sufficient specificity for GDM diagnosis in isolation, necessitating the inclusion of additional biomarkers. Furthermore, the lack of standardized assays and reference ranges for these cytokines during pregnancy remains a limitation, which could restrict their application in clinical practice.

To date there is no evidence concerning the variation of IL-6 and TNF-α throughout the pregnancy, as there are no longitudinal studies. However, in our review we included studies that targeted in different trimesters in pregnancy, as shown in Table 1. Most studies included data from univariate analyses, and they do not indicate a potential exacerbation throughout the course of pregnancy. Hence, the actual effect of confounding factors may be true and particularly significant. As a result, further longitudinal studies are needed to validate this hypothesis.

While observational studies have identified significant associations between elevated cytokine levels and subsequent GDM development, there remains a lack of large-scale, prospective validation studies confirming their predictive value in diverse populations. Moreover, IL-6 and TNF-α are infrequently integrated into multivariate risk models alongside traditional clinical and biochemical predictors, making it difficult to assess their additive utility in refining early screening approaches. Additionally, many studies assess these markers at a single time point, limiting our understanding of their longitudinal dynamics and predictive accuracy over the course of gestation.

These limitations highlight the need for standardized protocols, better-designed prospective cohorts, and integrated risk models to clarify the clinical relevance of IL-6 and TNF-α in the prediction and diagnosis of GDM.

## 6. Future Perspectives

The integration of biomarkers such as IL-6 and TNF-α into GDM risk assessment models holds significant potential for enhancing both diagnosis and treatment strategies. However, the clinical applicability of these biomarkers, especially when combined with traditional maternal demographic and metabolic data, remains an area that requires thorough investigation. Randomized controlled trials are essential to determine whether incorporating IL-6 and TNF-α measurements into clinical practice can lead to better outcomes in managing GDM. These trials are necessary to establish strong evidence regarding the effectiveness of IL-6 and TNF-α panels in informing clinical decision-making. RCTs could examine whether early detection of high-risk pregnancies, based on elevated IL-6 and TNF-α levels, leads to reduced GDM incidence, improved maternal and fetal health outcomes, and a reduction in complications such as macrosomia, preeclampsia, and cesarean sections. Moreover, RCTs can evaluate whether early interventions, guided by the inclusion of these biomarkers in risk assessments, result in measurable improvements in treatment outcomes. The use of IL-6 and TNF-α panels in routine GDM risk assessments may have substantial implications for healthcare costs. RCTs are needed to assess the cost-effectiveness of incorporating these biomarkers into clinical practice, by comparing the costs of these tests and associated interventions with their potential benefits (e.g., reduced long-term maternal health risks, fewer pregnancy complications, and improved post-pregnancy outcomes), to determine whether their widespread use is economically viable. One of the significant potential advantages of early intervention based on IL-6 and TNF-α levels is the potential improvement in long-term maternal health, particularly in reducing the risk of type 2 diabetes. RCTs could assess whether women identified as high-risk for GDM due to elevated levels of these biomarkers experience improved long-term metabolic outcomes, including a decreased incidence of type 2 diabetes or other chronic conditions. Additionally, RCTs must address the ethical considerations of early inflammatory marker testing and the risk of unnecessary treatments or interventions. These trials could help mitigate concerns regarding over-diagnosis or misclassification of women at risk, ensuring that interventions are only provided to those who will benefit. Furthermore, trials conducted across diverse populations are essential to evaluate whether the utility of IL-6 and TNF-α markers is applicable across various ethnic groups and socio-economic backgrounds, which may have differing baseline risks for GDM.

## 7. Conclusions

Gestational diabetes mellitus remains a significant obstetric and public health concern due to its association with both immediate pregnancy complications and long-term metabolic consequences for mothers and offspring. The recognition of inflammation as a contributor to insulin resistance and metabolic dysfunction during pregnancy accentuates the need to revisit traditional paradigms of GDM diagnosis and management. Among the various inflammatory mediators, IL-6 and TNF-α have demonstrated consistent associations with early pregnancy glycemic alterations and have shown promise as predictive biomarkers of GDM prior to the onset of hyperglycemia detectable by oral glucose tolerance testing. Their mechanistic involvement in disrupting insulin signaling, via serine phosphorylation of IRS-1, inhibition of GLUT4 translocation, and modulation of adipokine activity, further supports their biological plausibility.

Heterogeneity in study designs, assay techniques, and population characteristics currently limits the clinical applicability of these biomarkers. Standardization of sampling protocols, identification of gestational age-specific cut-off values, and integration into multivariable predictive models are essential next steps. As our understanding of the immunometabolic pathways in GDM deepens, IL-6 and TNF-α may serve not only as diagnostic adjuncts but also as potential targets for early therapeutic intervention. Future prospective cohort studies and randomized controlled trials are needed to validate their utility in personalized risk stratification, with the ultimate goal of improving maternal and fetal outcomes.

## Figures and Tables

**Table 1:** Overview of reported associations between IL-6/TNF-α and GDM across studies.

Study	Population Type	Population Size	Time	TNF-α (pg/mL)	IL-6 (ng/L)
Omazic et al., 2024 [36]	pregnant women	34 healthy pregnant women as a control group 13 pregnant women with GDM 14 pregnant women diagnosed with a glucose disorder in the first trimester	First trimester	decreased *p* = 0.64	increased *p* = 0.37
Muthuraman et al., 2024 [37]	Pregnant women	27 Controls vs. 27 GDM	24–28 weeks	decreased	N/A
Wang et al., 2020 [35]	Pregnant women	68 Controls vs. 74 GDM	24–28 weeks	increased *p* = 0.001	N/A
Tenenbaum-Gavish et al., 2020 [38]	Pregnant women	185 Controls vs. 20 GDM	First trimester	decreased *p* = 0.037	increased *p* = 0.524
Chiti et al., 2020 [39]	Pregnant women	25 Controls vs. 25 GDM	Third trimester	decreased *p* = 0.900	N/A
Siddiqui et al., 2019 [34]	Pregnant women	50 Controls vs. 53 GDM	12 to 26 weeks	N/A	increased *p* = 0.001
Mohammed et al., 2018 [40]	Pregnant women	100 Controls vs. 100 GDM	24–28 weeks	increased *p* < 0.05	N/A
Zhang et al., 2017 [41]	Pregnant women	60 Controls vs. 60 GDM	24–28 weeks	increased *p* = 0.32	increased *p* = 0.002

N/A: not applicable.
